# A combination of anti‐PD‐L1 mAb plus Lm‐LLO‐E6 vaccine efficiently suppresses tumor growth and metastasis in HPV‐infected cancers

**DOI:** 10.1002/cam4.1143

**Published:** 2017-08-09

**Authors:** Po‐Lin Lin, Ya‐Min Cheng, De‐Wei Wu, Yu‐Ju Huang, Hun‐Chi Lin, Chih‐Yi Chen, Huei Lee

**Affiliations:** ^1^ Graduate Institute of Cancer Biology and Drug Discovery Taipei Medical University Taipei Taiwan; ^2^ Department of Obstetrics and Gynecology National Cheng Kung University Hospital Tainan Taiwan; ^3^ Global BioPharma, Inc. Taipei Taiwan; ^4^ Department of Surgery Chung Shan Medical Hospital Taichung Taiwan

**Keywords:** Anti‐PD‐L1 mAb, HPV, Lm‐LLO‐E6 vaccine, NSCLC

## Abstract

PD‐1/PD‐L1 immunotherapy is viewed as having clinical benefits in advanced cancers but is effective in only a few patients, suggesting that an efficient combination approach is needed to improve efficacy. Immunohistochemistry analysis indicated that PD‐L1 expression was correlated with the E6 expression in tumors from 122 lung cancer patients. The poorest survival occurred in PD‐L1‐positive/E6‐positive tumor. PD‐L1 expression was increased by the expression of E6, but not the E7, oncoprotein in lung and cervical cancer cells. PD‐L1 expression was responsible for E6‐mediated colony formation and soft agar growth. Therefore, PD‐L1 secreted from tumor cells may directly promote tumor progression, particularly in E6‐positive tumors. Immune deficiency nude mice were used to test the possibility that combining anti‐PD‐L1 mAb with Lm‐LLO‐E6 vaccine could have a higher antitumor activity compared with anti‐PD‐L1 mAb or Lm‐LLO‐E6 vaccine alone. A greater antitumor activity was obtained with anti‐PD‐L1 mAb + Lm‐LLO‐E6 vaccine than with anti‐PD‐L1 mAb or Lm‐LLO‐E6 alone in subcutaneous and metastatic tumors induced by TL‐1 and SiHa cells. The longest survival time for nude mice was observed in the anti‐PD‐L1 mAb + Lm‐LLO‐E6 vaccine group. In conclusion, an anti‐PD‐L1 mAb + Lm‐LLO‐E6 vaccine may be an efficient treatment for suppression of tumor growth and metastasis induced by HPV‐infected cells.

## Introduction

Program death ligand‐1 (PD‐L1) acts as an inhibitor of human T‐cell responses by binding to its receptor PD‐1 to create the tumor microenvironment. This, in turn, results in tumor progression due to tumor immune surveillance 1, 2. The PD‐L1 protein is abundantly expressed in various human cancers, including non‐small‐cell lung cancer (NSCLC) 3. PD‐L1‐positive lung tumors show significantly lower numbers of tumor infiltrating lymphocytes (TILs) when compared to PD‐L1‐negative lung tumors, which suggests that PD‐L1 expression in tumor cells may contribute to the negative regulation of the antitumor immune response in NSCLC 4. Furthermore, a high expression of PD‐L1 may contribute to poor prognosis and tumor immune escape by suppressing the maturation of tumor infiltrating dendritic cells 5.

Poor prognosis in NSCLC is associated with the epithelial‐mesenchymal transition (EMT), a key process that drives cancer metastasis 6, 7, 8. The EMT is highly associated with an inflammatory tumor microenvironment in NSCLC and immune activation that coexists with the elevation of multiple targetable immune checkpoint molecules, such as PD‐L1. A further association is seen with the increases in tumor infiltration by CD4 + Foxp3+ regulatory T cells that display an EMT phenotype 6. The PD‐1/PD‐L1 axis therefore plays a crucial role in tumor progression, the EMT, and poor prognosis in NSCLC.

Human papillomavirus (HPV) 16/18 infection is associated with lung cancer development in Taiwan 9, 10. The HPV16/18 E6 oncoprotein promotes tumor growth and invasion by attenuating the expression of IL‐10, TIMP‐3, paxillin, and FOXM1 11, 12, 13, 14. Tumor invasion induced by E6‐mediation of these molecules occurs by triggering the EMT 11, 12, 13, 14. We therefore speculated that the E6 oncoprotein might induce PD‐L1 expression, which would induce tumor invasion and confer poor prognosis in NSCLC.

Antibody‐mediated blockade of PD‐L1 can induce a durable tumor regression and prolonged stabilization of disease in patients with NSCLC 15. Our preliminary data showed a positive correlation between the HPV16/18 E6 oncoprotein and PD‐L1 expression in a small subset of NSCLC patients. An oncoprotein vaccine, the Lm‐LLO‐E7 vaccine, suppresses tumor growth in a TC‐1 animal model 16, 17. In this study, the Lm‐LLO‐E7 and Lm‐LLO‐E6 vaccines (obtained from Global BioPharma Inc.; Taipei, Taiwan) were used to verify whether a combination of anti‐PD‐L1 monoclonal antibody (mAb) + Lm‐LLO‐E6 vaccine might suppress tumor growth and metastasis more strongly in animal models injected with HPV16 E6‐positive TL‐1 lung cancer cells, when compared to antibody and vaccine therapies alone (i.e., anti‐PD‐L1 mAb, Lm‐LLO‐E6 vaccine, Lm‐LLO‐E7 vaccine, and anti‐PD‐L1 mAb + Lm‐LLO‐E7 combinations). SiHa cervical cancer cells positive for HPV16 served as the positive controls.

## Materials and Methods

### Study subjects

Lung tumor specimens were collected from 122 patients who underwent primary NSCLC surgical resection at the Department of Thoracic Surgery, Taichung Veterans General Hospital (Taichung, Taiwan) between 1998 and 2004. Patients were asked to submit written informed consent; the study was approved by the Institutional Review Board (TMUH No. 201301051). The tumor type and stage of each collected specimen were histologically determined in accordance with the World Health Organization classification system. Cancer relapse data were obtained from chart review and confirmed by thoracic surgeons. Clinical parameters and OS and relapse‐free survival (RFS) data were collected from chart review and the Taiwan Cancer Registry, Department of Health, Executive Yuan, ROC.

### Cell lines

TL‐1 and TL‐4 cells were cultured from the plural effusions from lung adenocarcinoma patients 10 and were kindly provided by Dr. Y.‐W. Cheng (Graduate Institute of Cancer Biology and Drug Discovery, Taipei Medical University, Taipei, Taiwan) 10. SiHa and C33A cells were obtained from the Bioresource Collection and Research Center, the Food Industry Research and Development Institute (Hsinchu, Taiwan). TL‐1, TL‐4, and C33A cancer cell lines were maintained in RPMI‐1640 medium (HyClone, Logan, UT). SiHa cancer cell lines were maintained in DMEM medium (HyClone, Logan, UT). The media contained 10% fetal bovine serum (FBS) supplemented with penicillin (100 U/mL) and streptomycin (100 mg/mL). The cells were cultured according to the suppliers' instructions. Once resuscitated, the cell lines were routinely authenticated (once every 6 months; the cells were last tested in December 2012) by cell morphology monitoring, growth curve analysis, species verification via isoenzymology and karyotyping, identity verification via short tandem repeat profiling analysis, and contamination checks.

### Immunohistochemistry analysis

Immunohistochemistry was performed to evaluate PD‐L1 protein expression. PD‐L1 antibody was purchased from Genetex (GTX104763, Irvine, CA). HPV E6 antibody was purchased from Santa Cruz Biotechnology (sc‐1583, Dallas, TX). Formalin‐fixed and paraffin‐embedded specimens were cut into 3 *μ*m sections, mounted on glass, and dried overnight at 37°C. All sections were then deparaffinized in xylene, rehydrated through alcohol, and washed in phosphate‐buffered saline. This buffer was used for all subsequent washes. Sections were heated in a microwave oven twice for 5 min in citrate buffer (pH 6.0) and then incubated for 60 min at room temperature following a conventional streptavidin peroxidase method (LSAB Kit K675, DAKO, Carpinteria, CA). Signals were developed with 3, 3′‐diaminobenzidine for 5 min and the sections were counterstained with hematoxylin. Negative controls were obtained by leaving out the primary antibody. The signal intensities were evaluated independently by three observers. Immunostaining scores were defined as the cell staining intensity (0 = nil; 1 = weak; 2 = moderate; and 3 = strong) multiplied by the percentage of labeled cells (0–100%), leading to scores from 0 to 300. A score greater than or equal to 150 was defined as “high” immunostaining, whereas a score <150 was defined as “low”.

### Plasmid construction, transfection, and stable clone selection

The HPV 16 E6 cDNA plasmid and HPV 16 E6/E7 small hairpin (sh)RNA were described previously 11. The target sequence of the PD‐L1 shRNA was GCT GCA CTA ATT GTC TAT TGG. The RNAi template was cloned into the vector pCDNA‐HU6 as described previously. Plasmids containing the PD‐L1 expression construct were constructed by cloning the full‐length human PD‐L1 cDNA (GenBank accession number NM_014143) into the pcDNA3.1 eukaryotic expression vector, which also expresses a neomycin (Neo) resistance gene. Nonspecific shRNA with a scrambled sequence and an empty vector expression were used as the controls in the knockdown and ectopic expressing experiments, respectively. The transfection and stable clone selection procedures have been described previously 18.

### Anchorage‐independent soft agar colony formation

Anchorage‐independent growth was assayed as the ability of cells to form colonies on soft agar plates. The bottom agar consisted of growth medium containing 10% fetal bovine serum and 0.5% agarose in six‐well plates. A total of 300 cells were resuspended in growth medium containing 10% fetal bovine serum and 0.4% agarose and placed on top of the bottom agar. The cells were grown at 37°C in a humidified incubator in 5% CO_2_. Colonies were visualized and quantified with a microscope after 14 days of cultivation, and the colonies larger than 100 *μ*m in diameter were counted.

### The subcutaneous animal model

Six‐ to eight‐week‐old female BALB/c nude mice and C57Bl/6 mice were purchased from National Laboratory Animal Center (Taipei, Taiwan). Mice were cared for under protocols approved by the NLAC Animal Care and Use Committee. Lm‐LLO‐E6 (E6) vaccine and Lm‐LLO‐E7 (E7) vaccine were injected intraperitoneally at doses of 5 × 10^6^ CFU/mouse. The anti‐PD‐L1 mAb obtained from BioLegend (San Diego, CA) was injected intravenously at a dose of 25 *μ*g/mouse. The aim of the experiment was to determine the tumor growth changes induced by different treatments when compared with the control group. Briefly, mice were subcutaneously implanted with 500,000 TL‐1 cells/mouse in the right flank on day 0. On day 7 (tumor size ~3–4 mm in diameter), the mice were injected with Lm‐LLO‐E6 or Lm‐LLO‐E7 intraperitoneally and/or with anti‐PD‐L1 mAb intravenously. Mice were treated with anti‐PD‐L1 mAb an additional three times, on days 14, 21, and 28 after tumor implantation. The control group of mice was only injected with TL‐1 or SiHa cells. Tumors were measured every 3–4 days using digital calipers, and tumor volume was calculated using the formula V = (W2 × L)/2, where V is volume, L is length (longer diameter), and W is width (shorter diameter). A flow chart is presented in Figure S1.

### The tail‐vein metastatic lung tumor animal model

Lm‐LLO‐E6 (E6) and Lm‐LLO‐E7 (E7) vaccines were injected intraperitoneally at a dose of 5 × 10^6^ CFU/mouse. The anti‐PD‐L1 mAb was injected intravenously at a dose of 25 *μ*g/mouse. The aim of the experiment was to determine the changes in tumor growth and survival induced by the different treatments. Briefly, mice were injected with 100,000 TL‐1 or SiHa cells/mouse by tail‐vein injection on day 0. On day 14, the mice were injected with Lm‐LLO‐E6 or Lm‐LLO‐E7 vaccine intraperitoneally and/or with anti‐PD‐L1 mAb intravenously. Mice were treated with anti‐PD‐L1 mAb an additional three times, on days 21, 28, 35 after tumor cell injections. Mice continued to eat normally until death. A flow chart is presented in Figure S1.

### Statistical analysis

All statistical analyses were conducted using the SPSS statistical software program, as described previously (version 17.0; SPSS, Inc., Chicago, IL) 18, 19. A two‐sided analysis of the variance in the statistical tests was conducted, and *P* < 0.050 was considered statistically significant.

## Results

### The expressions of E6 and PD‐L1 are positively correlated in lung tumors and are associated with poor prognosis in NSCLC patients

We collected 122 surgically resected lung tumors from NSCLC patients to examine whether E6 expression could be associated with PD‐L1 expression. The data for E6 expression were obtained from previous studies 11, 12, 13, 14. Immunohistochemistry analysis indicated that E6‐positive lung tumors exhibited a higher frequency of positive PD‐L1 expression (66.7% vs. 47.9%, *P* = 0.039; Table S1 and Figure S2). The PD‐L1 expression was not associated with clinical parameters such as age, gender, smoking status, and stage, but it was associated with tumor histology, indicating that positive PD‐L1 was expressed more frequently in adenocarcinoma than in squamous cell carcinoma (63.8% vs. 45.3, *P* = 0.042; Table S1).

**Figure 1:**
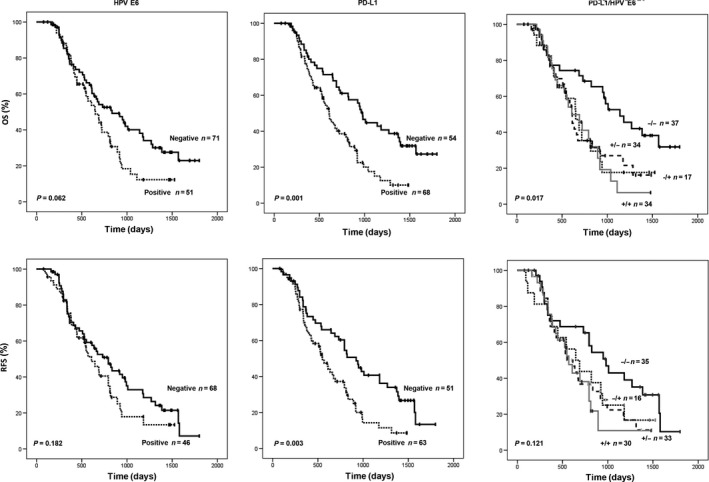
Kaplan–Meier survival analysis for assessing the influence of HPV16/18 E6 expression, PD‐L1 expression, and a combination of both expressions on overall survival (OS) and relapse‐free survival (RFS) patients in NSCLC.

The possible association between E6 and PD‐L1 expression, singly and combined, and overall survival (OS) and relapse‐free survival (RFS) was examined in NSCLC patients. Kaplan–Meier analysis showed that shorter OS and RFS were observed in patients with positive PD‐L1 tumors than with negative PD‐L1 tumors (*P* = 0.001 for OS, *P* = 0.003 for RFS; Fig. 1 middle panel), but the E6 oncoprotein showed no prognostic value in this study population (Fig. 1 left panel). However, the combination of PD‐L1 and E6 expression had a prognostic value for OS, but not for RFS (*P* = 0.017 for OS, *P* = 0.121 for RFS; Figure 1 right panel). Cox‐regression analysis further confirmed that patients with positive PD‐L1 tumors had shorter OS and RFS periods when compared to patients with negative PD‐L1 tumors (hazard ratio, HR, 1.69, 95% CI, 1.06–2.67, *P* = 0.026 for OS; HR, 1.55, 95% CI, 0.97–2.48, *P* = 0.045 for RFS; Table 1). The 5‐year survival percentage was lower in patients with positive PD‐L1 tumors than with negative PD‐L1 tumors (10.0 vs. 27.3% for OS, 8.6 vs. 13.3% for RFS; Table 1). However, the E6 oncoprotein showed no prognostic value in this study population. Interestingly, the PD‐L1‐positive/E6‐positive and PD‐L1‐positive/E6‐negative combinations showed prognostic value for OS and RFS when the PD‐L1‐negative/E6‐negative combination was used as the reference (Table 1). The lowest 5‐year survival percentage for OS and RFS was observed in patients with the high‐PD‐L1/E6‐positive combination when compared with other three combinations (6.4% for OS, 5.7% for RFS; Table 1). These results suggest that PD‐L1 expression may be used as an independent predictor of poor outcomes in HPV‐infected NSCLC.

**Table 1:** Cox regression analysis HPV E6, PD‐L1 status, and combining E6 with PD‐L1 status on OS and RFS in NSCLC patients

	Patient No.	OS					Patient No.	RFS				
		Median survival (month)	5‐year survival (%)	HR^a^	95%CI	*P*		Median months	5‐ year survival (%)	HR^a^	95%CI	*P*
E6 protein												
Negative	71	27.4	23.0	1			68	24.4	7.1	1		
Positive	51	21.5	12.3	1.49	0.95–2.32	0.080	46	19.0	13.4	1.28	0.80–2.03	0.302
PD‐L1												
Negative	54	32.1	27.3	1			51	27.4	13.3	1		
Positive	68	20.2	10.0	1.69	1.06–2.67	0.026	63	18.3	8.6	1.55	1.07–2.48	0.045
PD‐L1/HPV E6												
Negative/Negative	37	34.1	31.8	1			35	31.6	10.2	1		
Negative/Positive	17	21.5	17.6	1.89	0.84–3.81	0.076	16	21.5	16.7	1.45	0.71–2.99	0.312
Positive/Negative	34	20.3	16.1	2.00	1.08–3.71	0.027	33	18.3	11.2	1.71	0.93–3.15	0.086
Positive/Positive	34	19.0	6.4	2.30	1.23–4.30	0.009	30	18.3	5.7	1.90	0.99–3.65	0.053

The E6 immunostaining results were obtained from our previous reports 11, 12, 13, 14, and the detail procedure for the immunohistochemistry analysis was described previously.

a Adjusted for stage.

### The E6 oncoprotein increases PD‐L1 expression to promote colony formation and soft agar growth in HPV‐infected lung cancer cells

**Figure 2:**
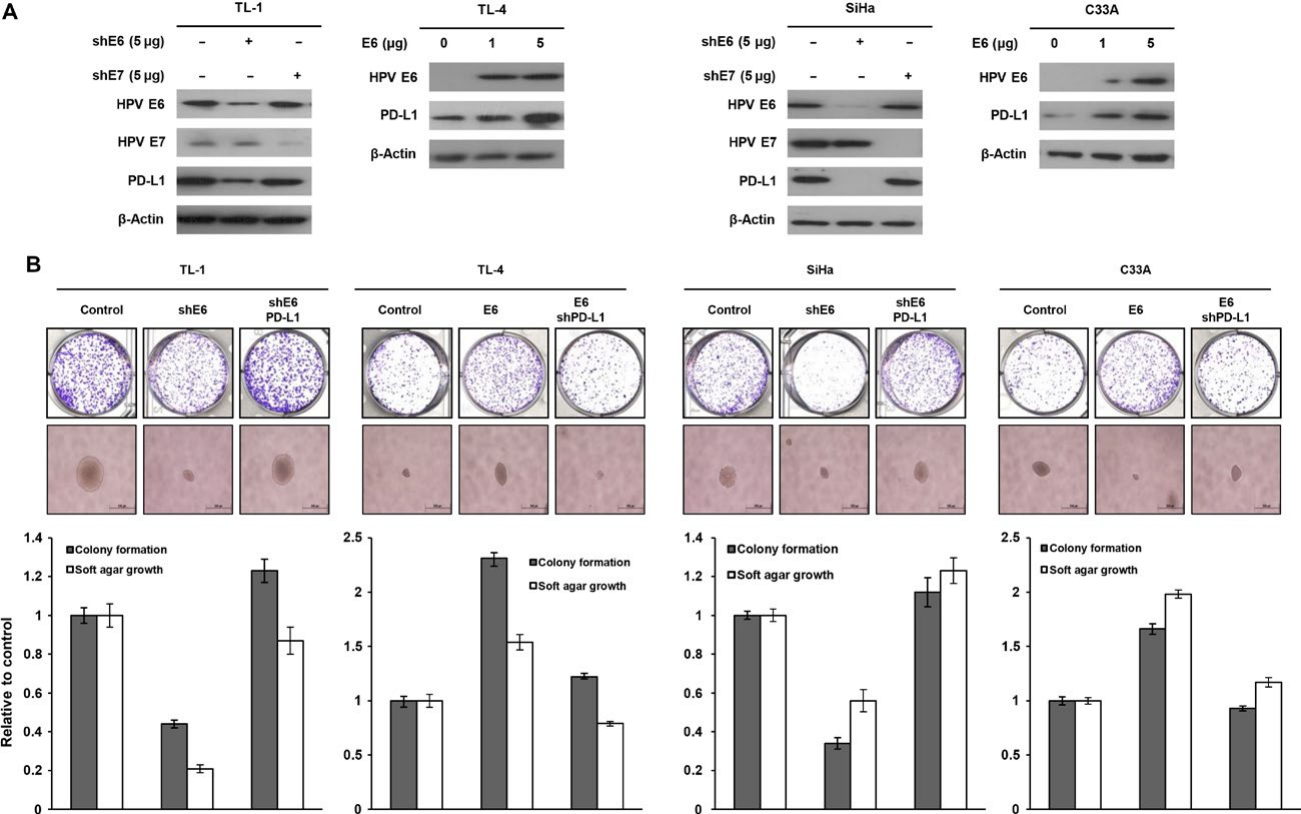
HPV16 E6‐mediated PD‐L1 expression may be responsible for colony formation and soft agar growth ability. (A) TL‐1 and SiHa cells were transfected with E6 shRNA or E7 shRNA; TL‐4 and C33A cells were transfected with an E6‐expressing plasmid for 48 h. Western blotting analysis was performed to evaluate the expression of E6, E7, and PD‐L1 in TL‐1 and TL‐4 cells. (B) TL‐1 and SiHa cells were transfected with E6 shRNA‐ and PD‐L1‐expressing plasmid; TL‐4 and C33A cells were transfected with E6‐expressing plasmid and PD‐L1 shRNA for 48 h. The colony formation ability was evaluated after 7 days. The soft agar growth ability was determined after 14 days.

We examined the possibility that E6 oncoprotein expression could increase PD‐L1 expression to promote colony formation and soft agar growth in HPV‐infected lung cancer cells. HPV16‐positive TL‐1 cells were collected for transfection with E6 or E7 small hairpin (sh)RNA and for comparison with HPV16‐negative TL‐4 cells transfected with the E6 or E7 expression vector. This comparison would verify whether the E6, but not the E7 oncoprotein, is responsible for PD‐L1 expression in lung cancer cells (Fig. 2A). Western blotting indicated that E6 and E7 protein expressions were decreased and increased as expected by E6 or E7 manipulation (Fig. 2A). Interestingly, PD‐L1 expression was markedly decreased by E6 knockdown in TL‐1 cells, but was increased by E6 overexpression in TL‐4 cells (Fig. 2A). The PD‐L1 expression was unchanged by E7 manipulation in both cell types (Fig. 2A). Similar responses were seen in the HPV16‐positive and HPV16‐negative cervical SiHa and C33A cells subjected to the same treatments (Fig. 2A). These results suggest that the E6, but not the E7, oncoprotein may be responsible for PD‐L1 expression in HPV‐infected lung cancer cells.

We next examined whether manipulation of PD‐L1 expression by E6 could attenuate the abilities for colony formation and soft agar growth in lung cancer cells. The representative colony growth in agar plates and soft agar growth colonies in soft agar plates are shown in Figure 2B (upper panel). The abilities of colony formation and soft agar growth were markedly decreased by E6 knockdown in TL‐1 cells, but both abilities were significantly increased by E6 overexpression in TL‐4 cells (Fig. 2B). However, the increase in the colony formation and soft agar growth ability by E6 oncoprotein was almost completely rescued by PD‐L1 knockdown in TL‐4 cells (Fig. 2B). Similar findings were observed in SiHa and C33A cervical cancer cells subjected to the same treatments (Fig. 2B). These results clearly indicate that E6‐mediated PD‐L1 expression may be responsible for colony formation and soft agar growth in HPV‐infected lung cancer cells.

### The anti‐PD‐L1 mAb+Lm‐LLO‐E6 vaccine almost completely suppresses subcutaneous tumor growth induced by TL‐1 cells in nude mice

**Figure 3:**
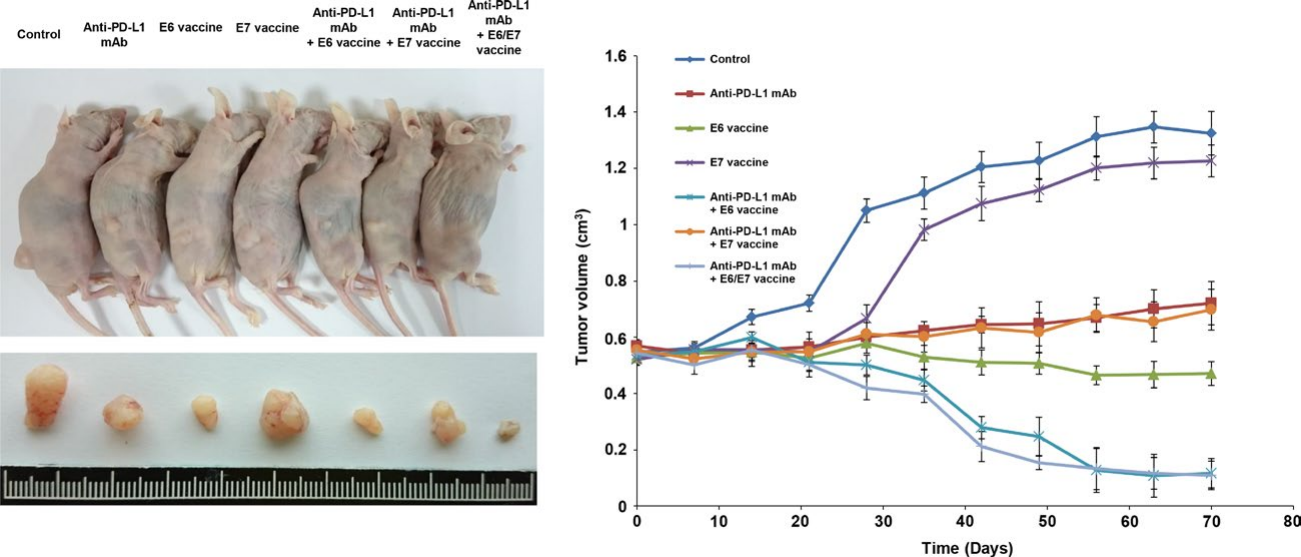
The combination of Lm‐LLO‐E6 vaccine + anti‐PD‐L1 mAb resulted in stronger suppression of subcutaneous tumor growth in nude mice. The nude mice were subcutaneously injected with HPV16‐infected TL‐1 cells that expressed E6 and E7 oncoprotein. After 7 days, the mice were treated with anti‐PD‐L1 mAb (25 *μ*g/mouse), Lm‐LLO‐E6 (E6) vaccine (5 × 106 CFU/mouse), Lm‐LLO‐E7 (E7) vaccine (5 × 106 CFU/mouse) or a combination of antibody plus one or both vaccines by peritoneal injection. The representative tumor burdens in the seven groups are illustrated. The tumor volume in the nude mice of each group was measured at 7‐day intervals from Day 0 to Day 70. Mean ± SEM values (cm^3^) were calculated from the tumor volumes of five nude mice in each group.

The anti‐PD‐L1 mAb may provide durable tumor suppression and a clinical benefit in advanced NSCLC 15. The finding that PD‐L1 may be responsible for E6‐mediated colony formation, soft agar growth, and poor prognosis in HPV‐infected lung cancer (Fig. 1), therefore suggested that the anti‐PD‐L1 mAb+Lm‐LLO‐E6 vaccine combination might have superior suppressive effects on tumor growth induced by TL‐1 cells in nude mice. The nude mice were randomly divided into seven groups of five nude mice each, which were injected with TL‐1 cells and then treated with either anti‐PD‐L1 mAb, Lm‐LLO‐E6 vaccine, Lm‐LLO‐E7 vaccine, anti‐PD‐L1 mAb+Lm‐LLO‐E6 vaccine, anti‐PD‐L1 mAb+Lm‐LLO‐E7 vaccine, or anti‐PD‐L1 mAb+Lm‐LLO‐E6/E7 vaccine. The control group mice were treated with TL‐1 cells only. The representative tumor burdens in each group are shown in Figure 3. The subcutaneous tumor burdens induced by TL‐1 cells were almost completely suppressed by anti‐PD‐L1 mAb + Lm‐LLO‐E6 vaccine or anti‐PD‐L1 mAb + Lm‐LLO‐E6/E7 vaccine when compared with the control group. However, a higher antitumor activity was observed in the Lm‐LLO‐E6 vaccine group than in the anti‐PD‐L1 mAb or the anti‐PD‐L1 + Lm‐LLO‐E7 vaccine groups. The tumors induced by TL‐1 cells were nearly unchanged by Lm‐LLO‐E7 vaccine treatment (Fig. 3). These results supported the findings of the cell models, indicating that PD‐L1 may be responsible for E6‐mediated tumor growth and metastasis in HPV‐infected cancers.

### Metastatic lung tumor nodules induced by TL‐1 cells in nude mice are efficiently suppressed by anti‐PD‐L1 mAb + Lm‐LLO‐E6 vaccine combination therapy

**Figure 4:**
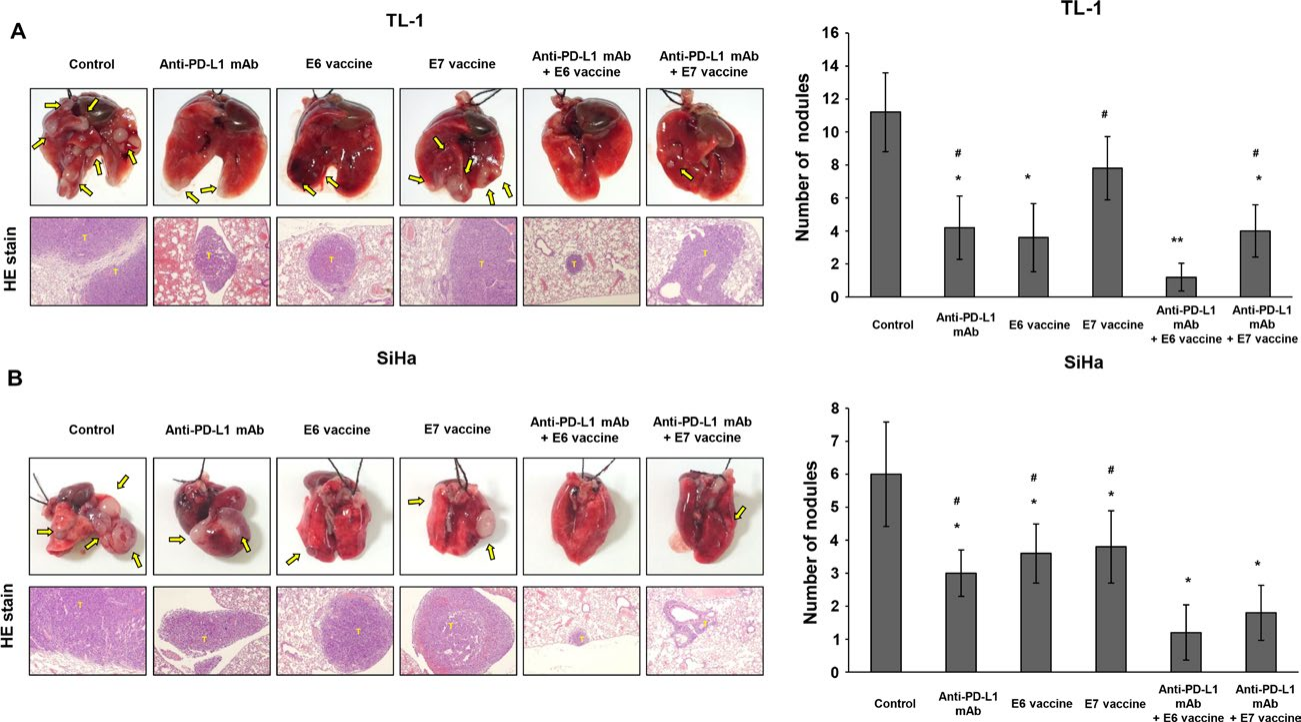
Metastatic lung tumor nodules induced by TL‐1 and SiHa cells in nude mice were efficiently suppressed by combined anti‐PD‐L1 mAb + Lm‐LLO‐E6 vaccine therapy. The nude mice were intravenously injected with HPV16‐infected (A) TL‐1 cells or (B) SiHa cells. After 14 days, the mice were treated with anti‐PD‐L1 mAb (25 *μ*g/mouse), Lm‐LLO‐E6 (E6) vaccine (5 × 106 CFU/mouse), Lm‐LLO‐E7 (E7) vaccine (5 × 106 CFU/mouse) or combinations of antibody and vaccine by peritoneal injection. Representative (H & E) staining is shown for the lung tumor nodules from each group of mice. The number of lung tumor nodules in each group of mice is also shown. Data represent means ± SD. The P value was statistically determined by the Student's *t*‐test. *P<0.05, compare with control; **P < 0.001, compared with control; ^#^P < 0.05 compared with Anti‐PD‐L1 mAb + Lm‐LLO‐E6 vaccine.

We also used a tail‐vein animal model to verify whether the anti‐PD‐L1 mAb + Lm‐LLO‐E6 combination can suppress metastatic lung tumor nodule formation induced by TL‐1 cells in nude mice. The experiments were halted at 77 days. The representative metastatic lung tumor nodules induced by TL‐1 cells in each group were compared with those induced by SiHa cells. Tumors were confirmed by H & E staining (Fig. 4A). Representative immunostaining results for the lung tumor nodules of each group are shown in Figure S3 and indicated a significant suppression of PD‐L1 and E6 expression in these lung tumor nodules by anti‐PD‐L1 mAb and Lm‐LLO‐E6 vaccine when compared with the control group. The number of metastatic lung tumor nodules induced by TL‐1 cells was most suppressed by the anti‐PD‐L1 mAb + Lm‐LLO‐E6 vaccine, followed by E6 vaccine, anti‐PD‐L1 mAb, anti‐PD‐L1 + Lm‐LLO‐E7 vaccine, and Lm‐LLO‐E7 vaccine when compared with the control group (Fig. 4A). Similar findings were observed in metastatic lung tumor nodules induced by SiHa cells in nude mice subjected to the same treatments (Fig. 4B).

**Figure 5:**
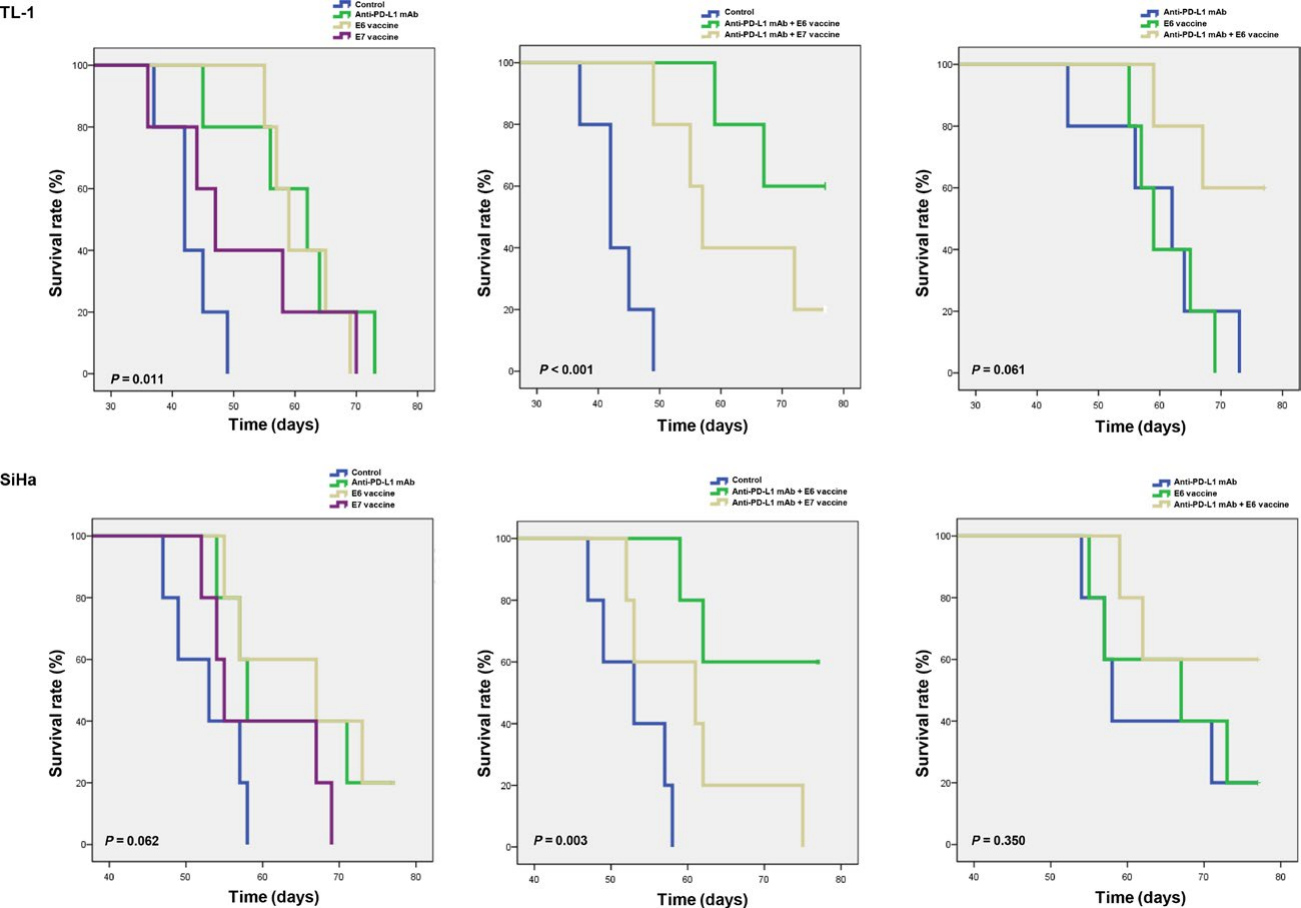
Kaplan–Meier analysis for assessing the influence of PD‐L1 mAb, Lm‐LLO‐E6 (E6) vaccine, Lm‐LLOE7 (E7) vaccine, the combination of PD‐L1 mAb+Lm‐LLO‐E6 vaccine, and the combination of PD‐L1 mAb + Lm‐LLO‐E7 vaccine on the survival of mice injected with TL‐1 or SiHa cells. The final day of the experiment was day 77.

The body weights of the control mice injected with TL‐1 cells were significantly lower than those of mice injected with anti‐PD‐L1 mAb, Lm‐LLO‐E6 vaccine, anti‐PD‐L1 mAb + Lm‐LLO‐E6 vaccine, and anti‐PD‐L1 mAb + Lm‐LLO‐E7 vaccine, but they did not differ from those that received Lm‐LLO‐E7 vaccine treatment (Fig. S4). Similar observations were made for the body weights of mice injected with SiHa cells subjected to the same treatments (Fig. S4). The survival days of the nude mice injected with TL‐1 or SiHa cells were significantly prolonged by the anti‐PD‐L1 mAb+Lm‐LLO‐E6 vaccine combination when compared with the control group (Fig. 5). Three of five mice were alive at 77 days in the anti‐PD‐L1 mAb + Lm‐LLO‐E6 vaccine and the anti‐PD‐L1 mAb + Lm‐LLO‐E7 vaccine groups treated with TL‐1 and SiHa cells. The last mouse died at 49 and 58 days, respectively, in the control groups treated with TL‐1 and SiHa cells. The survival days of the nude mice injected with TL‐1 cells were significantly prolonged with the anti‐PD‐L1 mAb + Lm‐LLO‐E6 vaccine when compared with the anti‐PD‐L1 mAb + Lm‐LLO‐E7 vaccine, Lm‐LLO‐E6 vaccine or anti‐PD‐L1 mAb (*P* = 0.004 for anti‐PD‐L1 mAb + Lm‐LLO‐E7 vaccine, *P* = 0.037 for Lm‐LLO‐E6 vaccine, *P* = 0.043 for anti‐PD‐L1 mAb, Fig. 5). These results suggest that the anti‐PD‐L1 mAb + Lm‐LLO‐E6 vaccine has the greatest potential to suppress metastatic lung tumor nodule formation in HPV‐infected lung and cervical cancer and, consequently, to prolong the survival of these mice.

## Discussion

The prevalence of HPV16/18 DNA and E6 oncoprotein expression were approximately 55% and 28% 9. In addition, the involvement of HPV16/18 infection in lung tumorigenesis was partially through p53 inactivation 10, increased hTERT 20 and IL‐10 expression 11, and decreased TIMP‐3 expression in HPV16‐infected TL‐1, TL‐2, or TL‐3 cells 12. However, the observations in a high prevalence of HPV16/18 infection and the involvement of HPV in lung tumorigenesis was rarely reported in other geographic areas 21, 22. The different causal association of HPV infections in lung cancer from different geographic areas should be further investigated.

The signaling pathways involved in the regulation of PD‐L1 expression in lung cancer cells have been studied previously 23, 24. For example, upregulation of PD‐L1 by EGFR activation through ERK/c‐Jun, but not through the AKT/S6 signaling pathway, mediates the immune escape in EGFR‐driven NSCLC 23. The induction of PD‐L1 expression by the EML4‐ALK oncoprotein and downstream MEK/ERK and PI3K/AKT signaling pathways was shown in NSCLC 24. The MEK/ERK and EGFR/PI3K/AKT signaling pathway may be activated by the E6 oncoprotein in NSCLC cells 25, 26. PD‐L1 expression in TL1 and SiHa cells was almost completely eliminated by tyrosine kinase inhibitor gefitinib, MEK/ERK inhibitor AZD6244 and NF‐*κ*B inhibitor BAY11‐7082, but unchanged by PI3K/AKT inhibitor wortamannin (Fig. S5). We therefore expected that PD‐L1 expression could be mediated by the E6 oncoprotein in NSCLC cells via the MEK/ERK and EGFR/ERK/NF‐*κ*B signaling pathways, but this requires further investigation. Nevertheless, to the best of our knowledge, this is the first report that the E6 oncoprotein promotes PD‐L1 expression in HPV‐infected lung and cervical cancer cells (Fig. 2).

The Lm‐LLO‐E7 vaccine was developed by Paterson's group, who reported that this vaccine suppressed tumor growth induced by TC‐1 cells in C57BL/6 mice via modulation of immune cells 16, 17. A clinical trial of Lm‐LLO‐E7 vaccine confirmed its safety for administration for treatment of late‐stage invasive carcinoma of the cervix 27. The mouse TC‐1 lung cancer cells were established by Dr. T.C. Wu (John Hopkin University) using a combined transfection of E6/E7 expression vectors. We also performed a TC‐1 animal model to verify whether the Lm‐LLO‐E7 vaccine could significantly suppress tumor growth induced by TC‐1 cells in C57BL/6 mice. The representative tumor burdens in the seven groups of mice are shown in Figure S6. The tumor burdens induced by TC‐1 cells in C57BL/6 mice were almost completely suppressed by the Lm‐LLO‐E7 vaccine, the combined anti‐PD‐L1 mAb + Lm‐LLO‐E7 vaccine, or the combined anti‐PD‐L1 mAb + Lm‐LLO‐E6/E7 vaccine when compared with the control group. However, the tumor burdens induced by TC‐1 cells in C57BL/6 mice were only moderately suppressed by the Lm‐LLO‐E6 vaccine, anti‐PD‐L1 mAb, or anti‐PD‐L1 mAb + Lm‐LLO‐E6 vaccine combination (Fig. S6). These results were consistent with Paterson's study 16, 17 and indicated that the Lm‐LLO‐E7 vaccine plays a critical role in suppression of TC‐1 cell‐induced tumor growth in C57BL/6 mice via suppression of T‐cell immunity and myeloid dendritic cell function 16, 17.

In this study, the tumor burdens induced by the human TL‐1 lung cancer cells in nude mice were almost completely suppressed by the anti‐PD‐L1 mAb + Lm‐LLO‐E6 vaccine combination but were nearly unchanged by the Lm‐LLO‐E7 vaccine (Fig. 3). The extremely different effects of the Lm‐LLO‐E6 and Lm‐LLO‐E7 vaccines on the TC‐1 and TL‐1 animal models reveal that tumors induced by endogenous HPV16‐infected TL‐1 cells differ from those induced by an engineered mouse TC‐1 cells. Moreover, this is the first indication that the Lm‐LLO‐E6 vaccine exhibits a greater potential than the Lm‐LLO‐E7 vaccine for suppression of tumor growth and metastasis in a human HPV16‐infected TL‐1 animal model. More importantly, the antitumor activity of Lm‐LLO‐E6 plus anti‐PD‐L1 mAb vaccine might be not through modulating PD‐L1‐mediated tumor immune surveillance because nude mice are almost immune deficient (Fig. S7). Immunostaining results from animal models seemed to suggest that Lm‐LLO‐E6 vaccine could neutralize E6 oncoprotein to inhibit PD‐L1 expression and consequently to suppress tumor growth and metastasis in nude mice induced by TL‐1 cells (Fig. S3). Therefore, the anti‐PD‐L1 mAb+Lm‐LLO‐E6 vaccine combination might be useful in a clinical trial for HPV‐infected lung cancer.

Women with HPV16‐positive grade 3 vulvar intraepithelial neoplasia can be treated by vaccination with a synthetic long‐peptide vaccine against the HPV16 E6 and E7 oncoproteins 28. Clearance of persistent HPV infection and cervical lesions can be achieved by a therapeutic DNA vaccine in cervical intraepithelial neoplasia 3 (CIN3) patients 29. Interestingly, this study strongly supports a previous study indicating that the anti‐PD‐L1 mAb significantly increases the therapeutic efficacy of Lm‐LLO immunotherapy 30. The most efficient suppression of tumor growth and metastasis induced by TL‐1 cells in nude mice was the anti‐PD‐L1 mAb + Lm‐LLO‐E6 vaccine combination, when compared with anti‐PD‐L1 mAb or Lm‐LLO‐E6 vaccine alone (Figs. 3 and 4).

In summary, we have provided evidence that an anti‐PD‐L1 mAb+Lm‐LLO‐E6 vaccine combination is an efficient therapeutic approach against HPV‐infected NSCLC. In addition, the anti‐PD‐L1 mAb + Lm‐LLO‐E6 vaccine combination also efficiently suppresses SiHa cell‐induced tumors in nude mice. Therefore, we suggest that anti‐PD‐L1 mAb + Lm‐LLO‐E6 vaccine combination therapy may have a greater clinical benefit than anti‐PD‐L1 or HPV DNA vaccine immunotherapy in cancer patients with HPV‐infected tumors.

## Conflict of Interest

The authors disclose no conflicts of interests.

## Supporting information


**Table S1.** Relationships of PD‐L1 expression with clinicopathological parameters in NSCLC cancer patients.
**Figure S1.** The experimental designs for evaluating the antitumor activity of anti‐PD‐L1 mAb, Lm‐LLO‐E6, Lm‐LLO‐E7, and both combinations in tumors induced by TL‐1 and SiHa cells in subcutaneous and tail‐vein nude mice models.
**Figure S2.** Representative immunostaining results for PD‐L1 expression in tumors from NSCLC patients.
**Figure S3.** Representative immunostaining results for E6 and PD‐L1 expression in tumors induced by TL‐1 and SiHa cells in each group of mice as indicated (magnification, ×200).
**Figure S4.** Changes in body weight in mice injected with TL‐1 or SiHa cells and treated with PD‐L1 mAb, Lm‐LLO‐E6 vaccine, or Lm‐LLO‐E7 vaccine.
**Figure S5.** Tyrosine Kinase inhibitor (Gefitinib), MEK/ERK inhibitor (AZD6244), PI3K/AKT inhibitor (Wortamannin), and NF‐κB inhibitor (BAY 11‐7082) were treated with TL1 and SiHa cells for 48h.
**Figure S6.** Lm‐LLO‐E7 vaccine alone and the combination of anti‐PD‐L1 mAb+Lm‐LLO ‐E7 vaccine markedly suppress tumor growth induced by TC‐1 cells in C57B/L6 mice compared with other treatments. The mice were subcutaneously injected with HPV16‐infected mouse TC‐1 cells.
**Figure S7.** Mature infiltrating T lymphocytes in tumor of each group of nude mice were not detected in nude mice with different treatments.

## References

[1] Anagnostou, V. K. , and J. R. Brahmer . 2015 Cancer immunotherapy: a future paradigm shift in the treatment of non‐small cell lung cancer. Clin. Cancer Res. 21:976–984. 25733707 10.1158/1078-0432.CCR-14-1187

[2] Chen, L. , and X. Han . 2015 Anti‐PD‐1/PD‐L1 therapy of human cancer: past, present, and future. J. Clin. Investig. 125:3384–3391. 26325035 10.1172/JCI80011 PMC4588282

[3] Kerr, K. M. , M. S. Tsao , A. G. Nicholson , Y. Yatabe , I. I. Wistuba , F. R. Hirsch , et al. 2015 Programmed death‐ligand 1 immunohistochemistry in lung cancer: in what state is this art? J. Thorac.Oncol. 10:985–989. 26134220 10.1097/JTO.0000000000000526

[4] Konishi, J. , K. Yamazaki , M. Azuma , I. Kinoshita , H. Dosaka‐Akita , and M. Nishimura . 2004 B7‐H1 expression on non‐small cell lung cancer cells and its relationship with tumor‐infiltrating lymphocytes and their PD‐1 expression. Clin. Cancer Res. 10:5094–5100. 15297412 10.1158/1078-0432.CCR-04-0428

[5] Mu, C. Y. , J. A. Huang , Y. Chen , C. Chen , and X. G. Zhang . 2011 High expression of PD‐L1 in lung cancer may contribute to poor prognosis and tumor cells immune escape through suppressing tumor infiltrating dendritic cells maturation. Med. Oncol. 28:682–688. 20373055 10.1007/s12032-010-9515-2

[6] Lou, Y. , L. Diao , E. R. Cuentas , W. L. Denning , L. Chen , Y. H. Fan , et al. 2016 Epithelial‐mesenchymal transition is associated with a distinct tumor microenvironment including elevation of inflammatory signals and multiple immune checkpoints in lung adenocarcinoma. Clin. Cancer Res. 22:3630–3642. 26851185 10.1158/1078-0432.CCR-15-1434 PMC4947453

[7] Gao, D. , L. T. Vahdat , S. Wong , J. C. Chang , and V. Mittal . 2012 Microenvironmental regulation of epithelial‐mesenchymal transitions in cancer. Can. Res. 72:4883–4889. 10.1158/0008-5472.CAN-12-1223 PMC3649848 23002209

[8] Shih, J. Y. , and P. C. Yang . 2011 The EMT regulator slug and lung carcinogenesis. Carcinogenesis 32:1299–1304. 21665887 10.1093/carcin/bgr110

[9] Cheng, Y. W. , H. L. Chiou , G. T. Sheu , L. L. Hsieh , J. T. Chen , C. Y. Chen , et al. 2001 The association of human papillomavirus 16/18 infection with lung cancer among nonsmoking Taiwanese women. Cancer Res. 61:2799–2803. 11306446

[10] Cheng, Y. W. , M. F. Wu , J. Wang , K. T. Yeh , Y. G. Goan , H. L. Chiou , et al. 2007 Human papillomavirus 16/18 E6 oncoprotein is expressed in lung cancer and related with p53 inactivation. Cancer Res. 67:10686–10693. 18006810 10.1158/0008-5472.CAN-07-1461

[11] Sung, W. W. , Y. C. Wang , P. L. Lin , Y. W. Cheng , C. Y. Chen , T. C. Wu , et al. 2013 IL‐10 promotes tumor aggressiveness via upregulation of CIP2A transcription in lung adenocarcinoma. Clin. Cancer Res. 19:4092–4103. 23743567 10.1158/1078-0432.CCR-12-3439

[12] Wu, D. W. , L. H. Tsai , P. M. Chen , M. C. Lee , L. Wang , C. Y. Chen , et al. 2012 Loss of TIMP‐3 promotes tumor invasion via elevated IL‐6 production and predicts poor survival and relapse in HPV‐infected non‐small cell lung cancer. Am. J. Pathol. 181:1796–1806. 22982189 10.1016/j.ajpath.2012.07.032

[13] Wu, D. W. , Y. W. Cheng , J. Wang , C. Y. Chen , and H. Lee . 2010 Paxillin predicts survival and relapse in non‐small cell lung cancer by microRNA‐218 targeting. Can. Res. 70:10392–10401. 10.1158/0008-5472.CAN-10-2341 21159652

[14] Chen, P. M. , Y. W. Cheng , Y. C. Wang , T. C. Wu , C. Y. Chen , and H. Lee . 2014 Up‐regulation of FOXM1 by E6 oncoprotein through the MZF1/NKX2‐1 axis is required for human papillomavirus‐associated tumorigenesis. Neoplasia 16:961–971. 25425970 10.1016/j.neo.2014.09.010 PMC4240922

[15] Brahmer, J. R. , S. S. Tykodi , L. Q. Chow , W. J. Hwu , S. L. Topalian , P. Hwu , et al. 2012 Safety and activity of anti‐PD‐L1 antibody in patients with advanced cancer. The New England journal of medicine 366:2455–2465. 22658128 10.1056/NEJMoa1200694 PMC3563263

[16] Peng, X. , S. F. Hussain , and Y. Paterson . 2004 The ability of two Listeria monocytogenes vaccines targeting human papillomavirus‐16 E7 to induce an antitumor response correlates with myeloid dendritic cell function. J. Immunol. 172:6030–6038. 15128786 10.4049/jimmunol.172.10.6030

[17] Gunn, G. R. , A. Zubair , C. Peters , Z. K. Pan , T. C. Wu , and Y. Paterson . 2001 Two Listeria monocytogenes vaccine vectors that express different molecular forms of human papilloma virus‐16 (HPV‐16) E7 induce qualitatively different T cell immunity that correlates with their ability to induce regression of established tumors immortalized by HPV‐16. J. Immunol. 167:6471–6479. 11714814 10.4049/jimmunol.167.11.6471

[18] Sung, W. W. , Y. C. Wang , Y. W. Cheng , M. C. Lee , K. T. Yeh , L. Wang , et al. 2011 A polymorphic ‐844T/C in FasL promoter predicts survival and relapse in non‐small cell lung cancer. Clin. Cancer Res. 17:5991–5999. 21807637 10.1158/1078-0432.CCR-11-0227

[19] Wu, D. W. , W. S. Liu , J. Wang , C. Y. Chen , Y. W. Cheng , and H. Lee . 2011 Reduced p21(WAF1/CIP1) via alteration of p53‐DDX3 pathway is associated with poor relapse‐free survival in early‐stage human papillomavirus‐associated lung cancer. Clin. Cancer Res. 17:1895–1905. 21325288 10.1158/1078-0432.CCR-10-2316

[20] Cheng, Y. W. , T. C. Wu , C. Y. Chen , M. C. Chou , J. L. Ko , and H. Lee . 2008 Human telomerase reverse transcriptase activated by E6 oncoprotein is required for human papillomavirus‐16/18‐infected lung tumorigenesis. Clin. Cancer Res. 14:7173–7179. 19010833 10.1158/1078-0432.CCR-08-0850

[21] Anantharaman, D. , T. Gheit , T. Waterboer , G. Halec , C. Carreira , B. Abedi‐Ardekani , et al. 2014 No causal association identified for human papillomavirus infections in lung cancer. Cancer Res. 74:3525–3534. 24760422 10.1158/0008-5472.CAN-13-3548

[22] Hasegawa, Y. , M. Ando , A. Kubo , S. Isa , S. Yamamoto , K. Tsujino , et al. 2014 Human papilloma virus in non‐small cell lung cancer in never smokers: a systematic review of the literature. Lung Cancer 83:8–13. 24252423 10.1016/j.lungcan.2013.10.002

[23] Chen, N. , W. Fang , J. Zhan , S. Hong , Y. Tang , S. Kang , et al. 2015 Upregulation of PD‐L1 by EGFR activation mediates the immune escape in EGFR‐Driven NSCLC: Implication for optional immune targeted therapy for NSCLC patients with EGFR mutation. J. Thorac. Oncol. 10:910–923. 25658629 10.1097/JTO.0000000000000500

[24] Ota, K. , K. Azuma , A. Kawahara , S. Hattori , E. Iwama , J. Tanizaki , et al. 2015 Induction of PD‐L1 expression by the EML4‐ALK oncoprotein and downstream signaling pathways in non‐small cell lung cancer. Clin. Cancer Res. 21:4014–4021. 26019170 10.1158/1078-0432.CCR-15-0016

[25] Liu, F. , B. Lin , X. Liu , W. Zhang , E. Zhang , L. Hu , et al. 2016 ERK signaling pathway is involved in HPV‐16 E6 but not E7 oncoprotein‐Induced HIF‐1alpha protein accumulation in NSCLC cells. Oncol. Res. 23:109–118. 26931433 10.3727/096504015X14496932933610 PMC7838632

[26] Wu, H. H. , J. Y. Wu , Y. W. Cheng , C. Y. Chen , M. C. Lee , Y. G. Goan , et al. 2010 cIAP2 upregulated by E6 oncoprotein via epidermal growth factor receptor/phosphatidylinositol 3‐kinase/AKT pathway confers resistance to cisplatin in human papillomavirus 16/18‐infected lung cancer. Clin. Cancer Res. 16:5200–5210. 20959404 10.1158/1078-0432.CCR-10-0020

[27] Maciag, P. C. , S. Radulovic , and J. Rothman . 2009 The first clinical use of a live‐attenuated Listeria monocytogenes vaccine: a Phase I safety study of Lm‐LLO‐E7 in patients with advanced carcinoma of the cervix. Vaccine 27:3975–3983. 19389451 10.1016/j.vaccine.2009.04.041

[28] Kenter, G. G. , M. J. Welters , A. R. Valentijn , M. J. Lowik , D. M. Berends‐van der Meer , A. P. Vloon , et al. 2009 Vaccination against HPV‐16 oncoproteins for vulvar intraepithelial neoplasia. N. Engl. J. Med. 361:1838–1847. 19890126 10.1056/NEJMoa0810097

[29] Kim, T. J. , H. T. Jin , S. Y. Hur , H. G. Yang , Y. B. Seo , S. R. Hong , et al. 2014 Clearance of persistent HPV infection and cervical lesion by therapeutic DNA vaccine in CIN3 patients. Nat. Commun. 5:5317. 25354725 10.1038/ncomms6317 PMC4220493

[30] Mkrtichyan, M. , N. Chong , R. Abu Eid , A. Wallecha , R. Singh , J. Rothman , et al. 2013 Anti‐PD‐1 antibody significantly increases therapeutic efficacy of listeria monocytogenes (Lm)‐LLO immunotherapy. J. Immunother. Cancer. 1:15. 24829751 10.1186/2051-1426-1-15 PMC4019896

