# Supplementary material for: A combination of anti‐PD‐L1 mAb plus Lm‐LLO‐E6 vaccine efficiently suppresses tumor growth and metastasis in HPV‐infected cancers
Source: Cancer Med. 2017 Aug 9;6(9):2052–62. doi: 10.1002/cam4.1143 (PMC5603833; doi:10.1002/cam4.1143)
Supplement: Supplementary file 1 — Table S1. Relationships of PD‐L1 expression with clinicopathological parameters in NSCLC cancer patients. Figure S1. The experimental designs for evaluating the antitumor activity of anti‐PD‐L1 mAb, Lm‐LLO‐E6, Lm‐LLO‐E7, and both combinations in tumors induced by TL‐1 and SiHa cells in subcutaneous and tail‐vein nude mice models. Figure S2. Representative immunostaining results for PD‐L1 expression in tumors from NSCLC patients. Figure S3. Representative immunostaining results for E6 and PD‐L1 expression in tumors induced by TL‐1 and SiHa cells in each group of mice as indicated (magnification, ×200). Figure S4. Changes in body weight in mice injected with TL‐1 or SiHa cells and treated with PD‐L1 mAb, Lm‐LLO‐E6 vaccine, or Lm‐LLO‐E7 vaccine. Figure S5. Tyrosine Kinase inhibitor (Gefitinib), MEK/ERK inhibitor (AZD6244), PI3K/AKT inhibitor (Wortamannin), and NF‐κB inhibitor (BAY 11‐7082) were treated with TL1 and SiHa cells for 48h. Figure S6. Lm‐LLO‐E7 vaccine alone and the combination of anti‐PD‐L1 mAb+Lm‐LLO ‐E7 vaccine markedly suppress tumor growth induced by TC‐1 cells in C57B/L6 mice compared with other treatments. The mice were subcutaneously injected with HPV16‐infected mouse TC‐1 cells. Figure S7. Mature infiltrating T lymphocytes in tumor of each group of nude mice were not detected in nude mice with different treatments. [file CAM4-6-2052-s001.docx]

**A combination of anti-PD-L1 mAb plus Lm-LLO-E6 vaccine efficiently suppresses tumor growth and metastasis in HPV-infected cancers**

Po-Lin Lin *et al.*

Supplementary Table

| Supplementary Table 1. Relationships of PD-L1expression with clinico-pathological parameters in NSCLC cancer patients. | | | | | | |
| --- | --- | --- | --- | --- | --- | --- |
|  | | PD-L1 protein | | | |  |
| Characteristics | Patient No. | Negative(%) | | Positive(%) | | *P* value |
| **Total patients** | 122 | 54 | (44.3) | 68 | (55.7) |  |
| **Age** |  |  |  |  |  |  |
| ≤67 | 64 | 25 | (39.1) | 39 | (60.9) | 0.225 |
| >67 | 58 | 29 | (32.3) | 29 | (50.0) |  |
| **Gender** |  |  |  |  |  |  |
| Female | 43 | 17 | (39.5) | 26 | (60.5) | 0.428 |
| Male | 79 | 37 | (46.8) | 42 | (53.2) |  |
| **Smoke** |  |  |  |  |  |  |
| No | 73 | 31 | (42.5) | 42 | (57.5) | 0.626 |
| Yes | 49 | 23 | (46.9) | 26 | (53.1) |  |
| **Tumor type** |  |  |  |  |  |  |
| AD | 69 | 25 | (36.2) | 44 | (63.8) | 0.042 |
| SCC | 53 | 29 | (54.7) | 24 | (45.3) |  |
| **Stage** |  |  |  |  |  |  |
| I | 45 | 23 | (51.1) | 22 | (48.9) | 0.416 |
| II | 23 | 8 | (34.8) | 15 | (65.2) |  |
| III | 54 | 23 | (42.6) | 31 | (57.4) |  |
| **HPV E6** |  |  |  |  |  |  |
| Negative | 71 | 37 | (52.1) | 34 | (47.9) | 0.039 |
| Positive | 51 | 17 | (33.3) | 34 | (66.7) |  |

Supplementary Figures


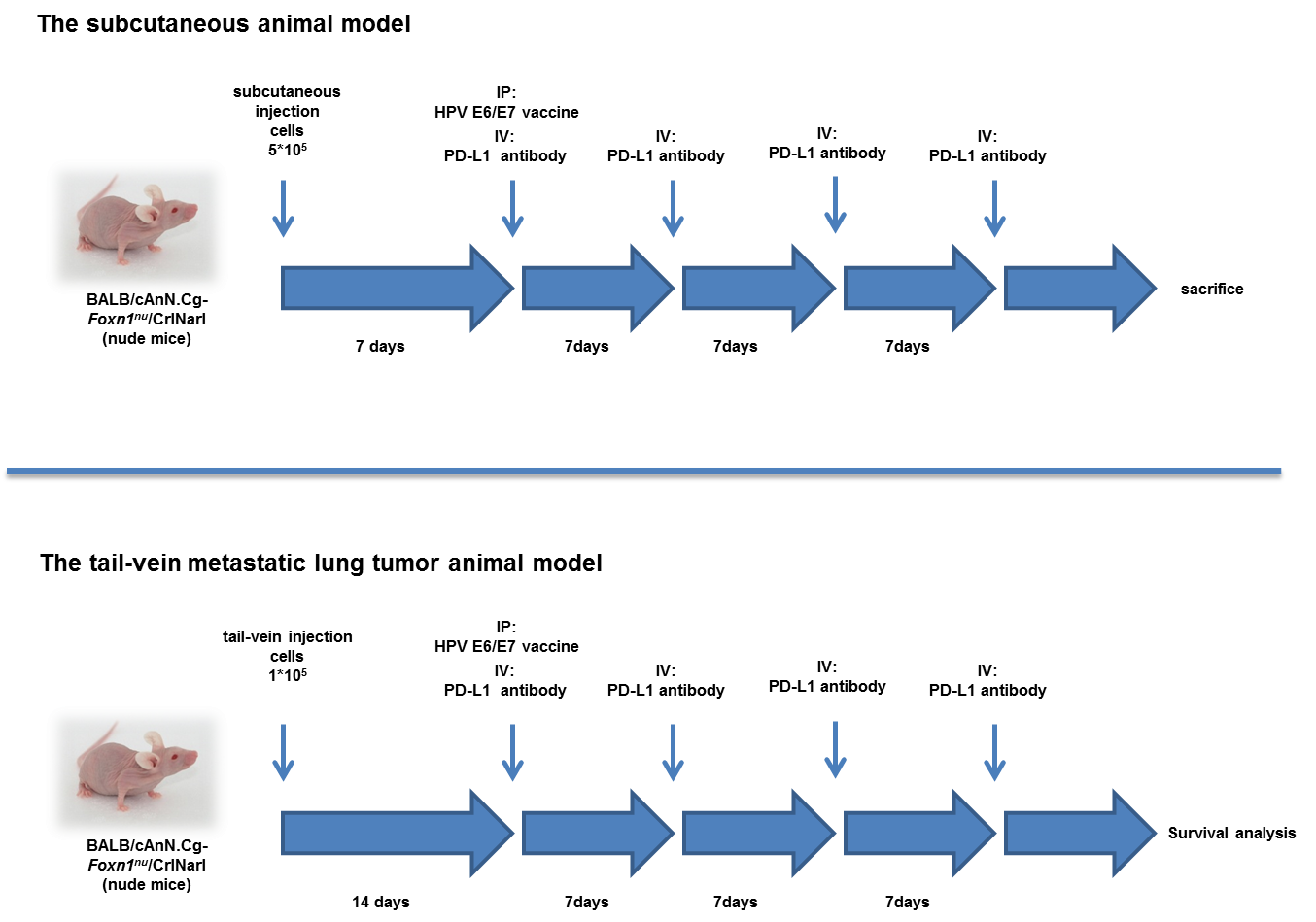


**Supplementary Figure 1.** The experimental designs for evaluating the antitumor activity of anti-PD-L1 mAb, Lm-LLO-E6, Lm-LLO-E7, and both combinations in tumors induced by TL-1 and SiHa cells in subcutaneous and tail-vein nude mice models.


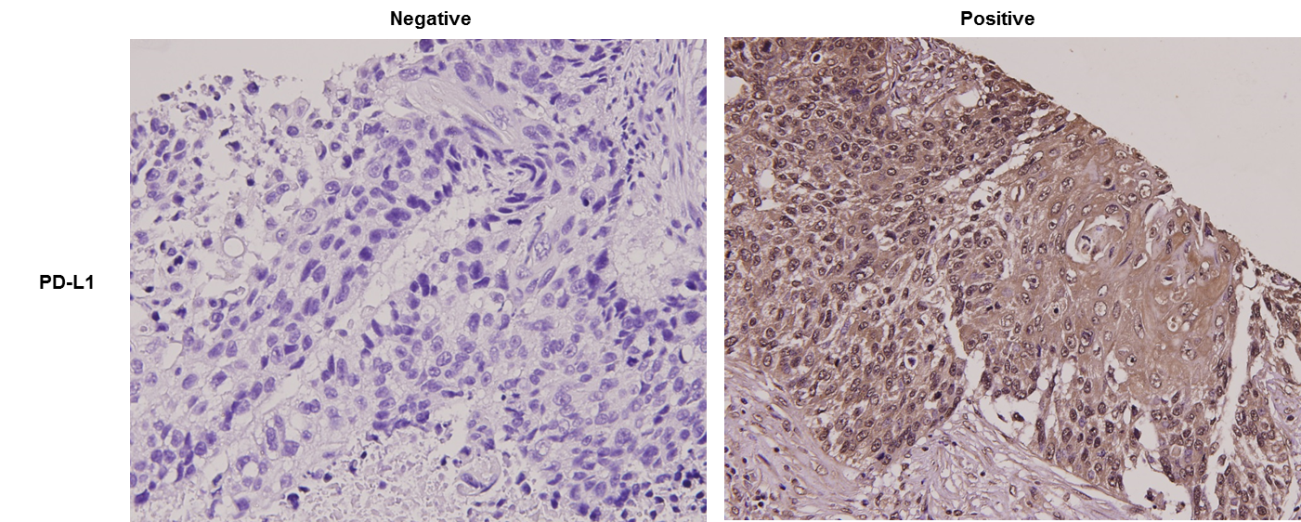


**Supplementary Figure 2.** Representative immunostaining results for PD-L1 expression in tumors from NSCLC patients.


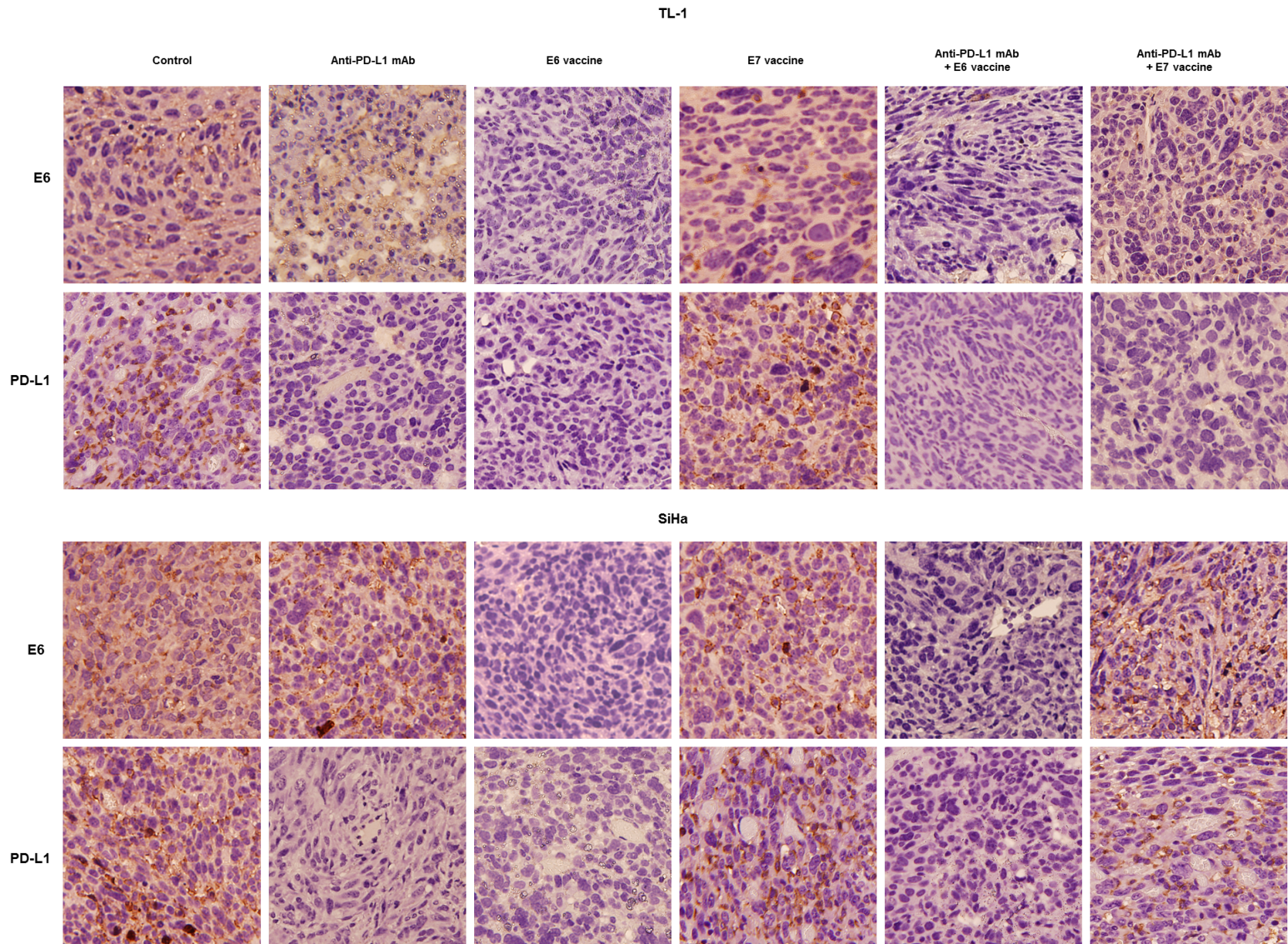


**Supplementary Figure 3.** Representative immunostaining results for E6 and PD-L1 expression in tumors induced by TL-1 and SiHa cells in each group of mice as indicated (magnification, ×200).


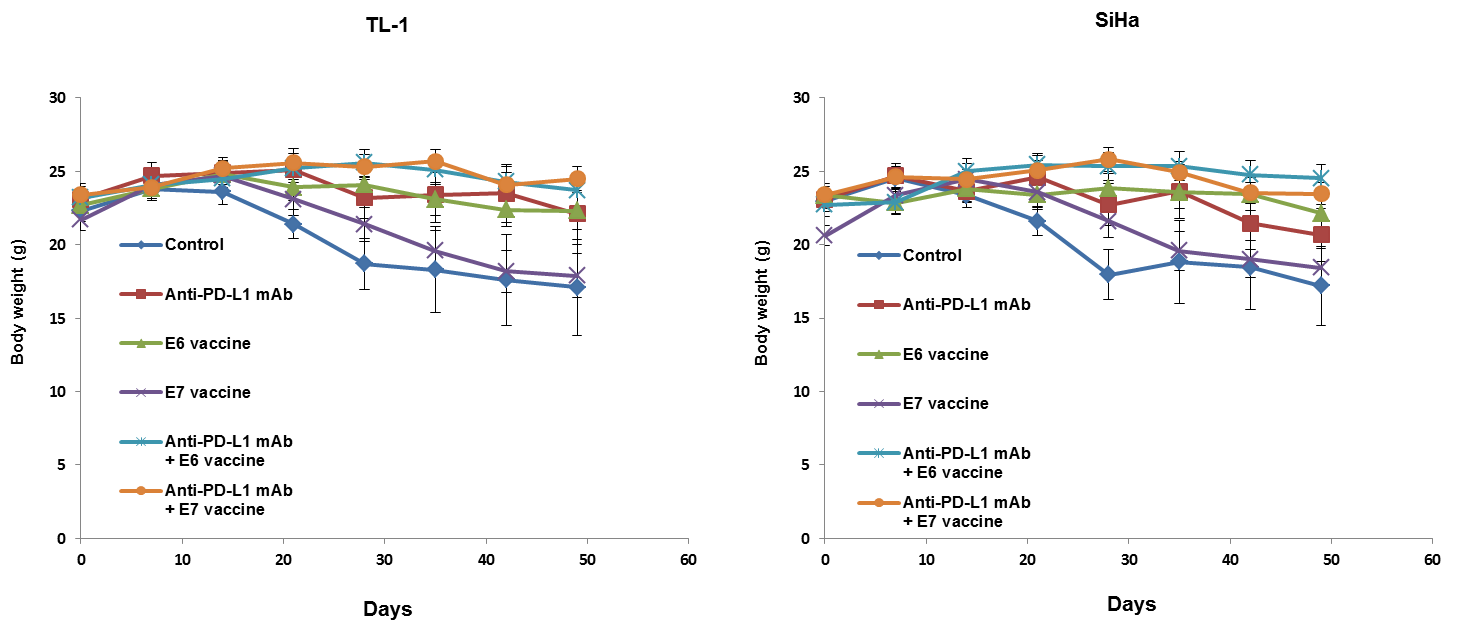


**Supplementary Figure 4.** Changes in body weight in mice injected with TL-1 or SiHa cells and treated with PD-L1 mAb, Lm-LLO-E6 vaccine, or Lm-LLO-E7 vaccine. The body weight was measured weekly throughout the experiment in mice injected with TL-1 or SiHa cells after treatment with PD-L1 mAb, Lm-LLO-E6 (E6) vaccine, Lm-LLO-E7 (E7) vaccine, PD-L1 mAb+Lm-LLO-E6 vaccine, and PD-L1 mAb+Lm-LLO-E7 vaccine. The P value was statistically calculated using the Student’s t-test.


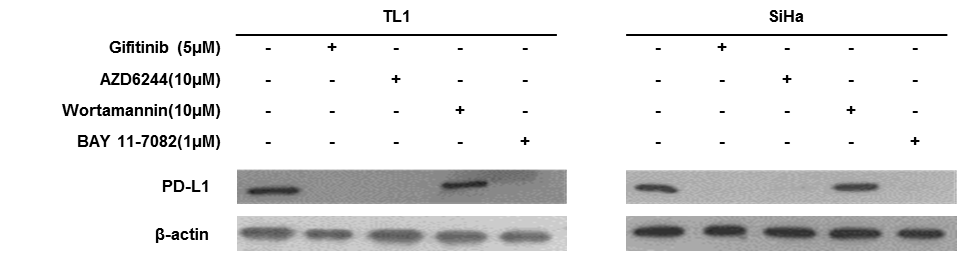


**Supplementary Figure 5.** Tyrosine Kinase inhibitor (Gefitinib), MEK/ERK inhibitor (AZD6244), PI3K/AKT inhibitor (Wortamannin) and NF-κB inhibitor (BAY 11-7082) were treated with TL1 and SiHa cells for 48h. The expression of PD-L1 changed by these inhibitors was evaluated by western blotting.


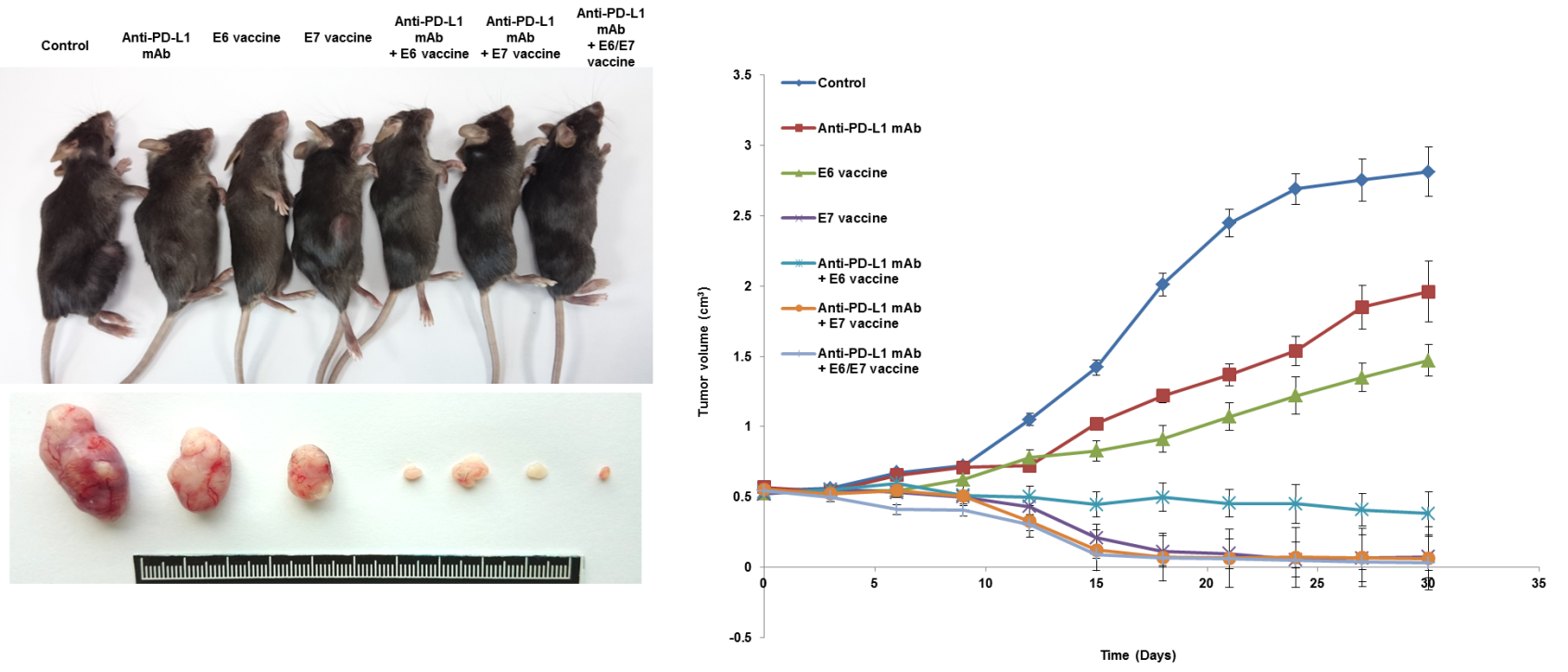


**Supplementary Figure 6.** Lm-LLO-E7 vaccine alone and the combination of anti-PD-L1 mAb+Lm-LLO-E7 vaccine markedly suppress tumor growth induced by TC-1 cells in C57B/L6 mice compared with other treatments. The mice were subcutaneously injected with HPV16-infected mouse TC-1 cells. After 7 days, the mice were treated with anti-PD-L1 mAb (25μg/mouse), Lm-LLo-E6 (E6) vaccine (5 × 10^6^ CFU/mouse), Lm-LLo-E7 (E7) vaccine (5 × 10^6^ CFU/mouse) and both combinations of antibody and vaccine by peritoneal injection. The representative tumor burdens in the seven groups are illustrated. The tumor volumes in the nude mice of each group were measured at 7-day intervals from Day 0 to Day 30. Mean ± S.E.M. values (cm^3^) were calculated from the tumor volume of five nude mice in each group.


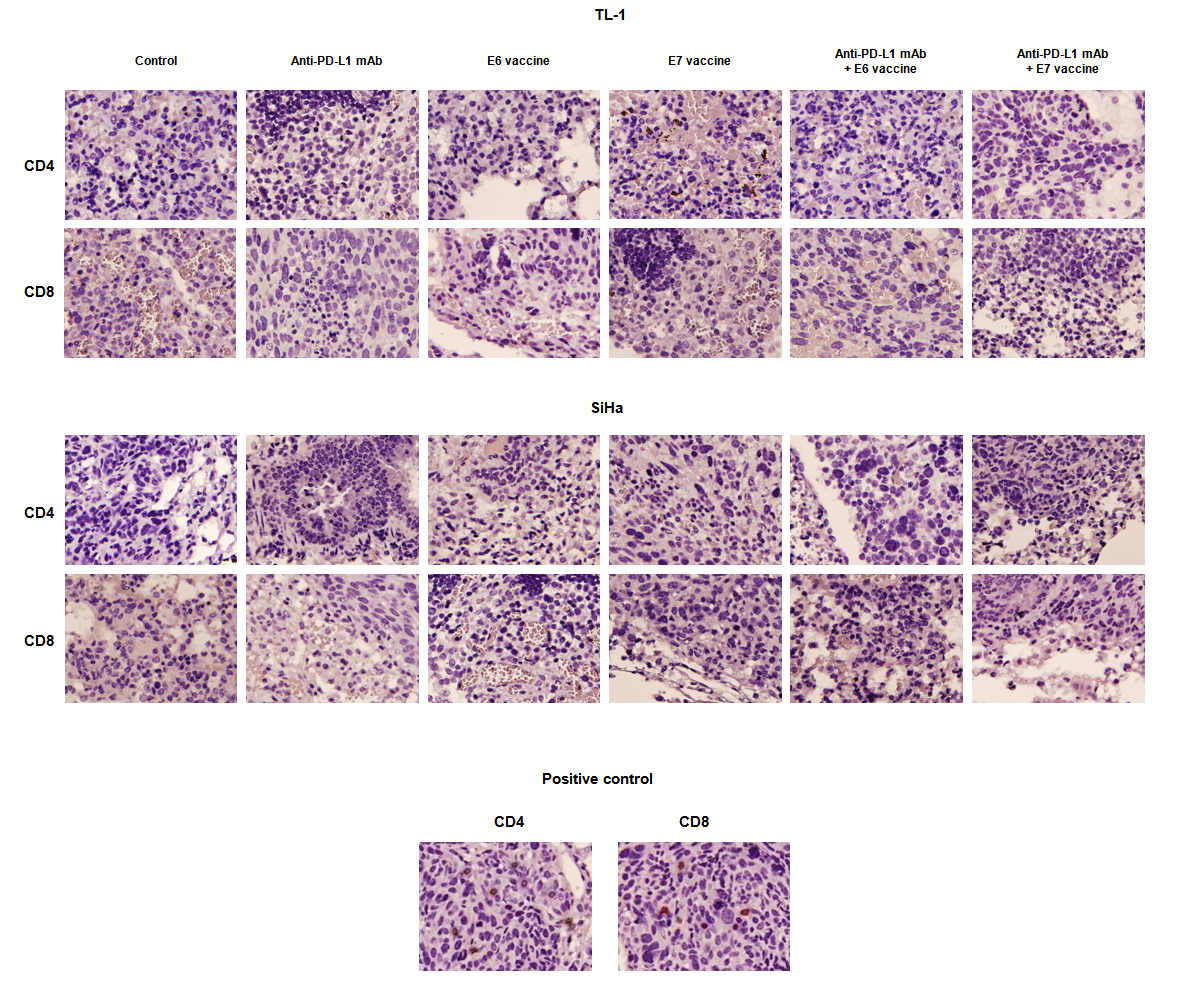


**Supplemenatary Figure 7.** Mature infiltrating T lymphocytes in tumor of each group of nude mice were not detected in nude mice with different treatments.
